# Supplementary material for: Collaborative intelligence in AI: Evaluating the performance of a council of AIs on the USMLE
Source: PLOS Digit Health. 2025 Oct 9;4(10):e0000787. doi: 10.1371/journal.pdig.0000787 (PMC12510544; doi:10.1371/journal.pdig.0000787)
Supplement: S2 File — (ZIP) [file pdig.0000787.s002.zip › S2 Data Raw data files containing unprocessed question inputs and LLM outputs including transcripts of deliberations/STEP3_Question-48-1_Type-MC-NJ.docx]

**Facilitator** **[Round 1]:** A 2-stage screening program for ovarian cancer is being designed. Which of the following sets of sensitivity and specificity is most likely preferred for Stage 1 and Stage 2 of the program? (A) Stage 1: High Sensitivity, Low Specificity; Stage 2: High Sensitivity, Low Specificity (B) Stage 1: High Sensitivity, Low Specificity; Stage 2: Low Sensitivity, High Specificity (C) Stage 1: Low Sensitivity, High Specificity; Stage 2: High Sensitivity, Low Specificity (D) Stage 1: Low Sensitivity, High Specificity; Stage 2: Low Sensitivity, Low Specificity

**AI_E:** RESTATING THE QUESTION: This question wants me to select the optimal sensitivity and specificity values for a 2-stage ovarian cancer screening program. To understand the question, sensitivity and specificity are two key test performance characteristics. A highly sensitive test decreases the chances of false negatives and thus, can detect almost all people with the disease. In contrast, a highly specific test decreases the chances of false positives, meaning it can correctly identify almost all people who do not have the disease. PLANNING: The assumptions here are that we desire the most effective screening program and that effectiveness is defined as catching as many cases as possible in the first stage (high sensitivity) and confirming these cases in the second stage (high specificity) to avoid wrongly labeling healthy individuals as having ovarian cancer. CLINICAL REASONING: In the first stage of screening, high sensitivity is preferred because we aim to identify as many true positives (people with ovarian cancer) as possible, without missing cases (minimizing false negatives). Thus, we may tolerate low specificity and potential false positives to not miss any potential cases of ovarian cancer. In the second stage, high specificity would be preferred as we aim to minimize any false positives from the first stage and ensure the individuals identified do genuinely have ovarian cancer. Therefore, low sensitivity here could be tolerated as fewer true cases are likely to be missed, having already been identified in the high sensitivity first stage. CONCLUDING: Considering the purpose of each stage in a sequential screening program, the first stage should ideally cast a wide net to capture all potential cases (high sensitivity), while the second stage should confirm those cases and avoid false alarms (high specificity). RESPONSE: With this reasoning, the most preferred set of sensitivity and specificity in a two-stage ovarian cancer screening program would be option (B) Stage 1: High Sensitivity, Low Specificity; Stage 2: Low Sensitivity, High Specificity.

**AI_A:** Restating the question: We're asked to choose the preferred sensitivity and specificity characteristics for the first and second stages of a two-stage ovarian cancer screening program. Sensitivity is the ability of a test to correctly identify individuals with a disease, while specificity is the capacity of a test to correctly identify individuals without the disease. Planning: We should consider the purpose of each stage in a multiple-step screening process. The primary aim of the first test in a screening program, which has a broader catchment, is to correctly identify as many positive cases as possible; hence, it needs to be highly sensitive. In contrast, the specificity of the initial stage is less of a concern, as any false positives can be ruled out with a subsequent test, preventing unnecessary treatment or invasive diagnostic procedures. For the second stage, a highly specific test would be more desirable to ensure that those without the disease are correctly identified and avoid unnecessary treatment or anxiety. Clinical Reasoning: Given these considerations, the first stage of screening should have high sensitivity to catch as many true positive cases as possible, meaning individuals with ovarian cancer are accurately identified, essentially reducing false negatives. Low specificity at this stage might raise the number of false positives, but it's acceptable in the first stage since those cases will further be scrutinized in the second stage. On the contrary, for the second stage, higher specificity is desirable. It results in fewer false positives, which means healthy individuals are less likely to be incorrectly diagnosed with the disease, preventing unnecessary treatments, interventions, and anxiety for these individuals. Concluding: The ideal combination of sensitivity and specificity for a two-stage ovarian cancer screening program would involve high sensitivity and low specificity for the first stage, and high specificity (irrespective of the sensitivity) for the second stage. Response: Therefore, the preferable set of sensitivity and specificity for this two-stage screening would be (B) Stage 1: High Sensitivity, Low Specificity; Stage 2: Low Sensitivity, High Specificity.

**AI_B:** RESTATING THE QUESTION: The question is asking us to determine the best sequencing of sensitivity and specificity for a two-stage ovarian cancer screening program. In medicine, sensitivity refers to the ability of a test to correctly identify patients who have a disease, whereas specificity refers to the test's ability to correctly identify patients who do not have the disease. PLANNING: Most 2-stage screening programs follow a rule that aims to maximize both the detection of true positive cases and the exclusion of true negative cases, while minimizing false positives and false negatives. This is usually accomplished by having a first stage with high sensitivity and a second stage with high specificity. By questioning this rule, we could generate the alternate assumption that another combination of stages might be better suited for the ovarian cancer screening. CLINICAL REASONING: The first stage of the screening program, with its high sensitivity, would catch as many true positive cases as possible, including those that are difficult to detect. This ensures that few cases of ovarian cancer are missed. However, a high sensitivity test often comes with a high rate of false positives, meaning the test might indicate that cancer is present when it is not. To counterbalance this, the second stage of the program should have high specificity. This would correctly identify those patients who do not have the disease, thereby screening out the false positives generated by the first stage. This two-stage approach ensures that as few cases of cancer as possible are missed while minimizing the number of healthy patients who are incorrectly identified as having the disease. CONCLUDING: Following the logic of clinical screening and diagnostic programs, the most preferred design for a two-stage ovarian cancer screening program would be one where the first stage has high sensitivity (maximizing the detection of true positive cases) and the second stage has high specificity (minimizing false positives). RESPONSE: Therefore, the appropriate choice reflecting this logic would be (B) Stage 1: High Sensitivity, Low Specificity; Stage 2: Low Sensitivity, High Specificity.

**AI_D:** RESTATING THE QUESTION: The query here is about the creation of a two-stage screening program for ovarian cancer. In this context, sensitivity refers to a test’s ability to correctly identify those with the disease, while specificity refers to the test’s ability to correctly pick out those without the disease. The question is asking which combination of sensitivity and specificity is most desirable for the first and second stages of the program. PLANNING: The identified assumptions here involve understanding the goals of each stage in a multi-stage screening program. Generally, the first stage of screening aims to cast a broad net and identify as many potential cases as possible - sensitivity is often a priority here. The second stage, following this, focuses on ruling out the false positives from the first stage, prioritizing specificity. The assumptions involve the principles of sensitivity and specificity being applied correctly across the two stages. CLINICAL REASONING: In the first stage of a screening program, we want to ensure we catch as many potential cases of the illness as possible, thus we require a high sensitivity. A low specificity at this stage is permissible as this means some healthy individuals may be incorrectly identified as potentially having the disease - these are our "false positives". For the second stage of the screening, our goal is to confirm the disease in those flagged by the first stage and we want to reduce the number of false positives. Thus, we require a test with high specificity to correctly identify those without the disease. CONCLUDING: By connecting these concepts, it becomes apparent that the optimal design of the screening program would potentially begin with a first stage of high sensitivity and low specificity - to ensure no potential cases are missed - and proceed with a second stage featuring low sensitivity and high specificity - to precisely confirm diagnoses and rule out false positives. RESPONSE: Therefore, the preferred option for the stages of the screening program for ovarian cancer is (B) Stage 1: High Sensitivity, Low Specificity; Stage 2: Low Sensitivity, High Specificity.

**AI_C:** RESTATING THE QUESTION: This question seems to focus on the design of a two-stage screening program for ovarian cancer. Such a program would involve two different tests or protocols, each with its own sensitivity and specificity. Sensitivity pertains to test's ability to correctly identify positive cases (a patient with the condition), while specificity concerns the test's ability to correctly identify negative cases (a patient without the condition). The optimal configuration of these characteristics for each stage of the program is the subject of inquiry. PLANNING: Now, let's consider the typical goals of a multi-stage cancer screening program. In the initial screening stage (Stage 1), the priority is usually to accurately identify as many cases of the disease as possible to ensure that very few cases are missed. This is best achieved with a test that has high sensitivity. However, this may also lead to some false positive results. Therefore, the second stage (Stage 2) should ideally have a high specificity to correct these false positives and confirm the true positive cases. CLINICAL REASONING: Applying our understanding of multi-stage screening programs, it's reasonable to infer that the first stage should use a test with high sensitivity. This will maximize the program's ability to pick up true positive cases, despite potentially resulting in some false positives. In the second stage, we can then check these preliminary positive cases with a highly specific test. A test with high specificity will be effective in determining that those without the disease are indeed disease-free, thus reducing the number of false positives. This combination would consequently optimize the detection and accurate diagnosis of ovarian cancer. CONCLUDING: Therefore, based on these considerations, it would be most preferable for a two-stage screening program for ovarian cancer to have Stage 1 with high sensitivity and low specificity, followed by Stage 2 with low sensitivity and high specificity. RESPONSE: Answer choice (B) Stage 1: High Sensitivity, Low Specificity; Stage 2: Low Sensitivity, High Specificity, is the most likely preferred set for the design of a two-stage ovarian cancer screening program.

**Facilitator** The consensus among the responses is that the optimal design for a two-stage ovarian cancer screening program would prioritize high sensitivity in the first stage and high specificity in the second stage. The first stage, with high sensitivity, aims to correctly identify as many individuals with the disease as possible, thereby minimizing false negatives. This stage may yield some false positives due to its low specificity, but these can be addressed in the second stage. The second stage, with high specificity, is designed to correctly identify individuals without the disease, thereby minimizing false positives. This stage may have low sensitivity, but this is less of a concern as most true cases would have been identified in the first stage. Therefore, the preferred set of sensitivity and specificity for this two-stage screening program would be (B) Stage 1: High Sensitivity, Low Specificity; Stage 2: Low Sensitivity, High Specificity.
